# Supplementary figures and images for: The Hunchback transcription factor determines interneuron molecular identity, morphology, and presynapse targeting in the Drosophila NB5-2 lineage
Source: PLoS Biol. 2025 Mar 31;23(3):e3002881. doi: 10.1371/journal.pbio.3002881 (PMC12135938; doi:10.1371/journal.pbio.3002881)

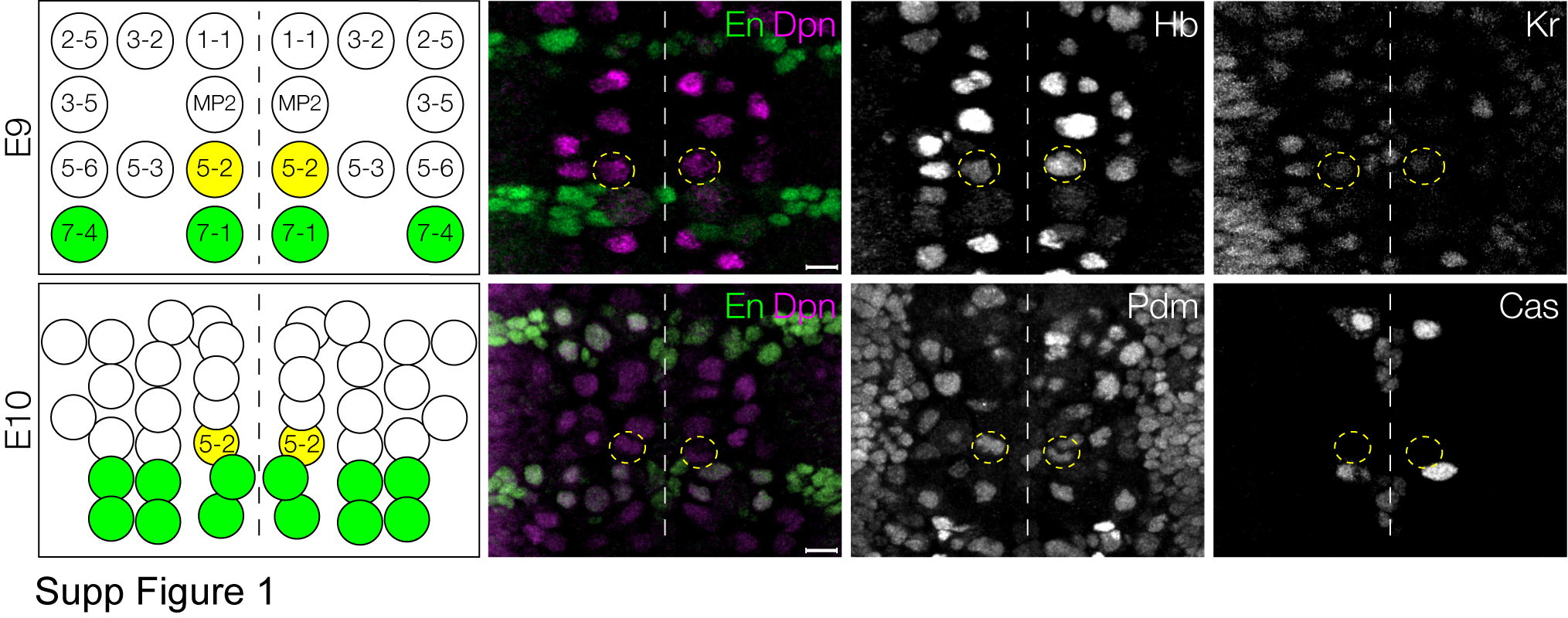

Supplement: S1 Fig — NB5-2 (yellow in schematic) was identified using the NB marker Dpn (magenta) and the row 6/7 expressing gene, Engrailed (En; green). NB5-2 was identified as the most medial Dpn+ /En-negative NB, anteriorly adjacent to the En domain. NB5-2 shows Hb/Kr expression in early stage 9 embryos (top panels) and Pdm expression by early stage10 (bottom panels). Scale bar: 5 µm. (TIF) [file pbio.3002881.s001.tif]

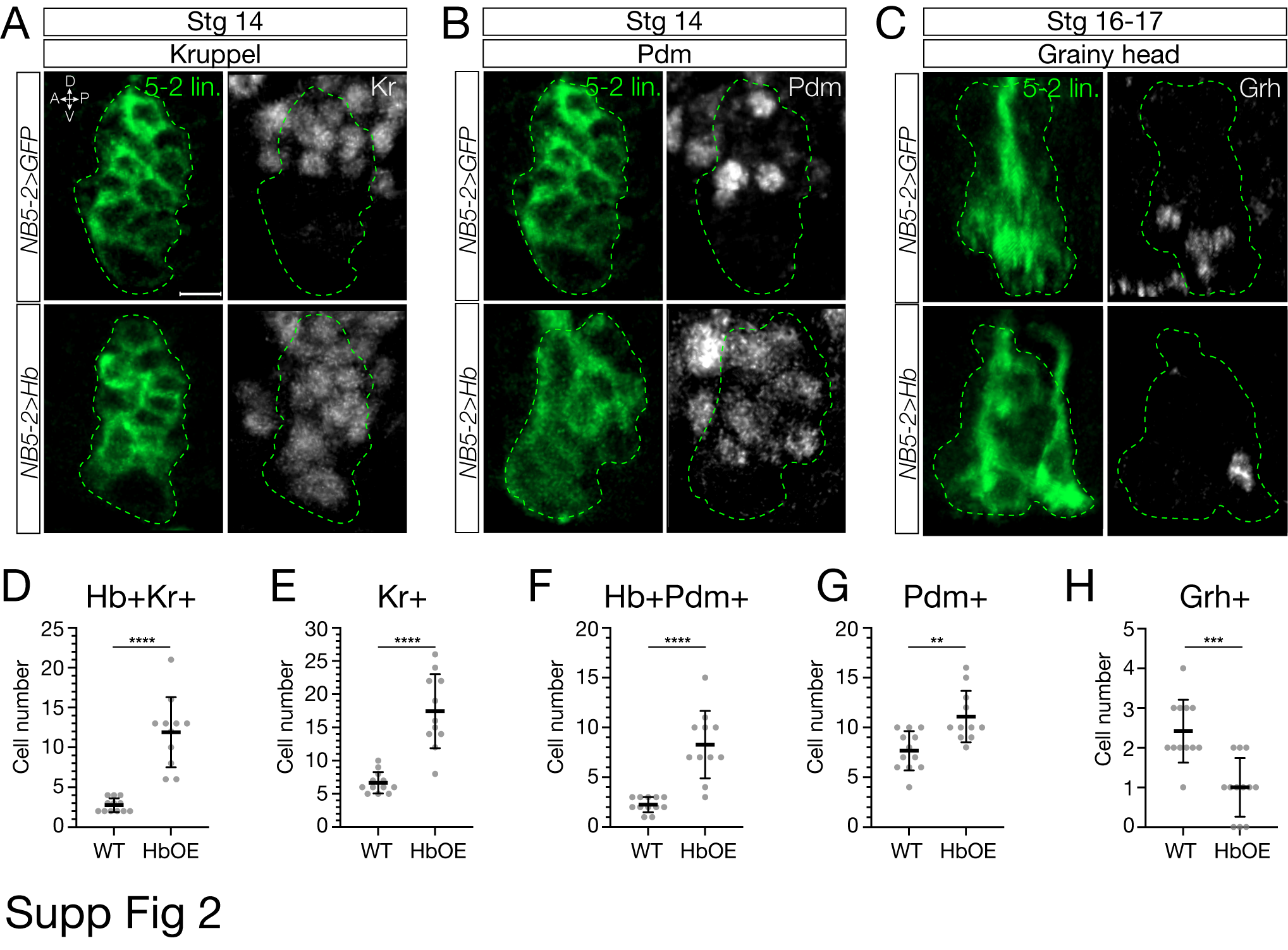

Supplement: S2 Fig — (A) Wildtype NB5-2 > GFP (green; top panels) and NB5-2 > Hb (bottom panels) progeny expressing Kr at stage 14. Anterior left, lateral view. Scale bar: 4 µm. (B) Wildtype NB5-2 > GFP (top panels) and NB5–2 > Hb (bottom panels) progeny expressing Pdm at stage 14. (C) Wildtype NB5–2 > GFP (top panels) and NB5–2 > Hb (bottom panels) progeny expressing Grh at stage 16–17. (D-H) Wild type and Hb overexpression quantified for the indicated markers; quantified in a minimum of 11 hemisegments from 3 embryos. The data underlying the graph in the figure can be found in S6 Data. (TIF) [file pbio.3002881.s002.tif]

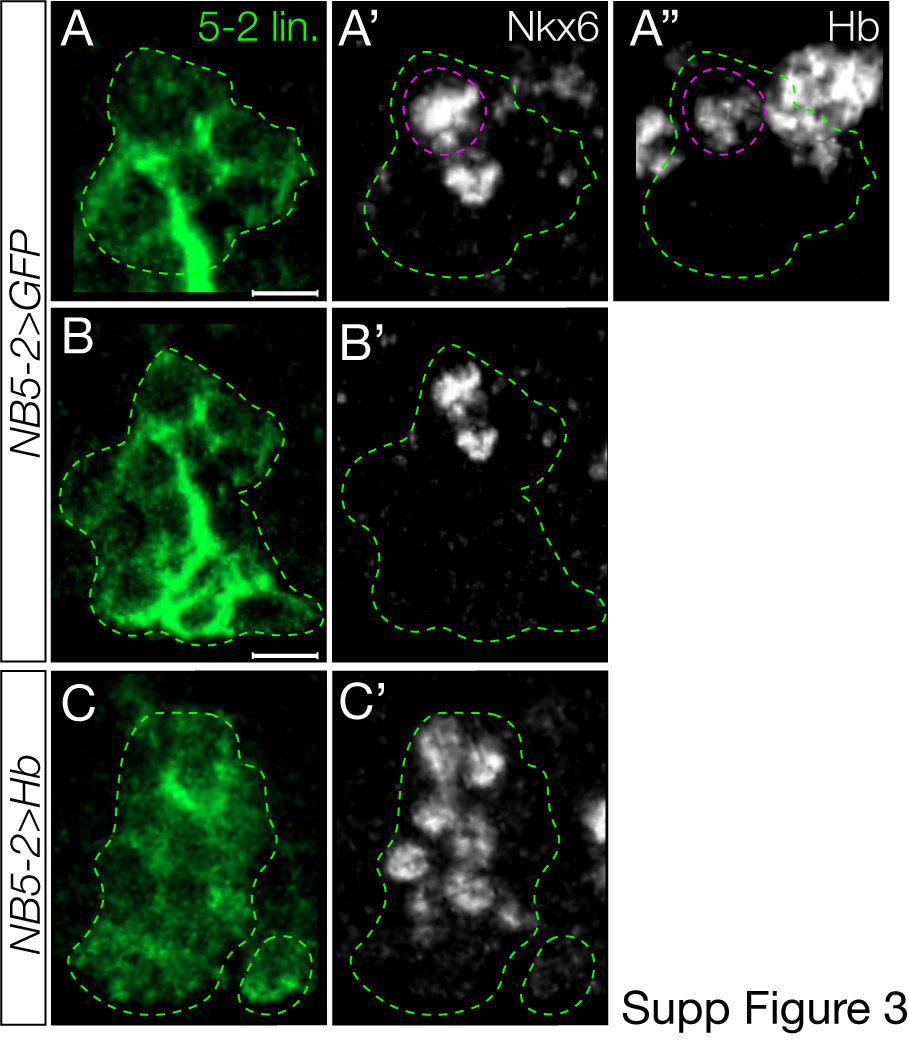

Supplement: S3 Fig — (A–B′) WT NB5-2 > GFP progeny (green, dotted outline) showing co-expression of Hb and the early-born marker Nkx6 at stage 17. Anterior left, lateral view. Scale bars: 4 µm. (C–C′) NB5-2 > Hb progeny results in an increase in Nkx6 neurons. (TIF) [file pbio.3002881.s003.tif]

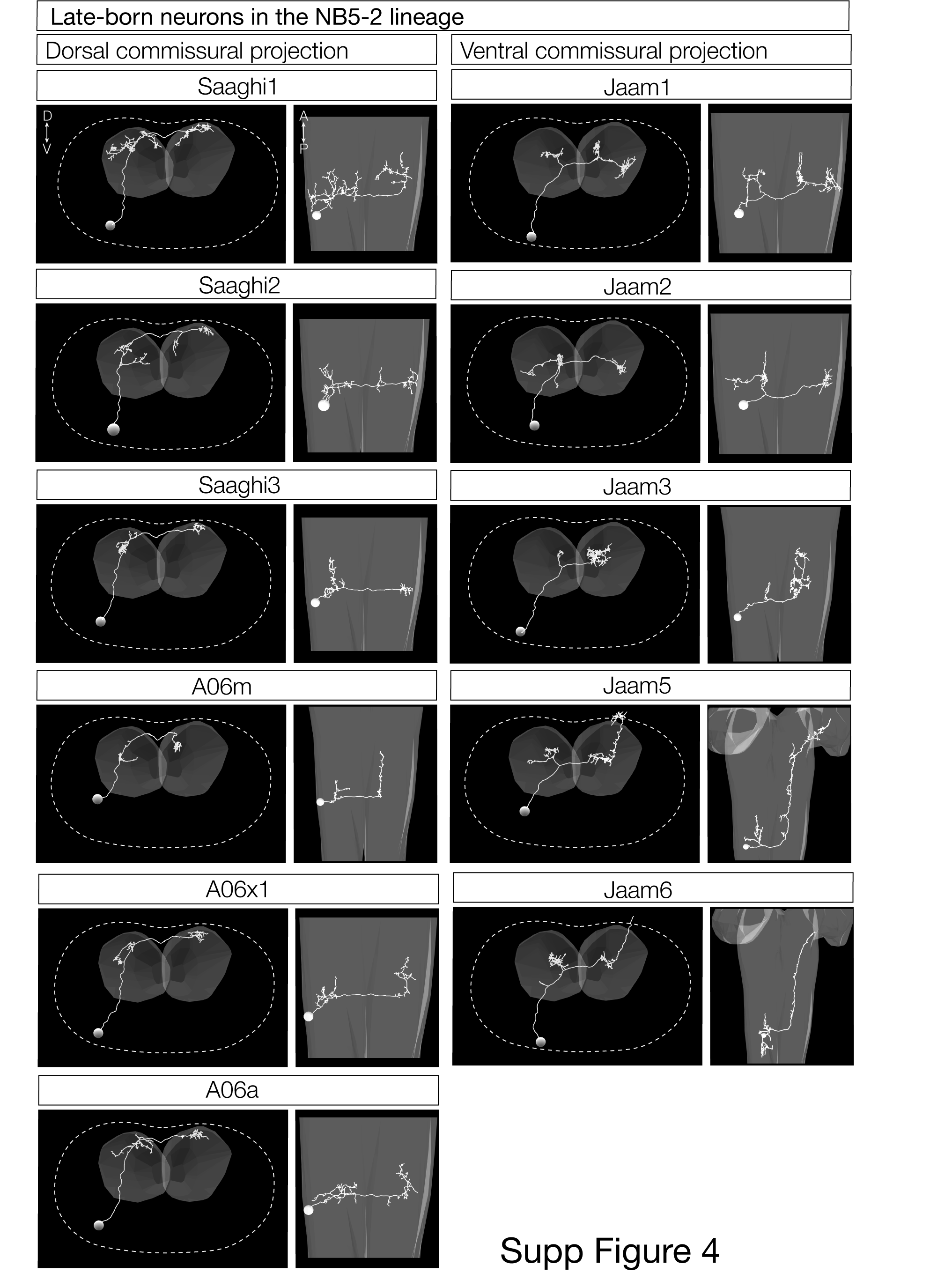

Supplement: S4 Fig — TEM reconstruction of wildtype NB5-2 late-born neurons organized by dorsal commissural projections (left column) and ventral commissural projections (right column). Dorsal up, posterior view (left panel); Anterior up, ventral view (right panel). (TIF) [file pbio.3002881.s004.tif]

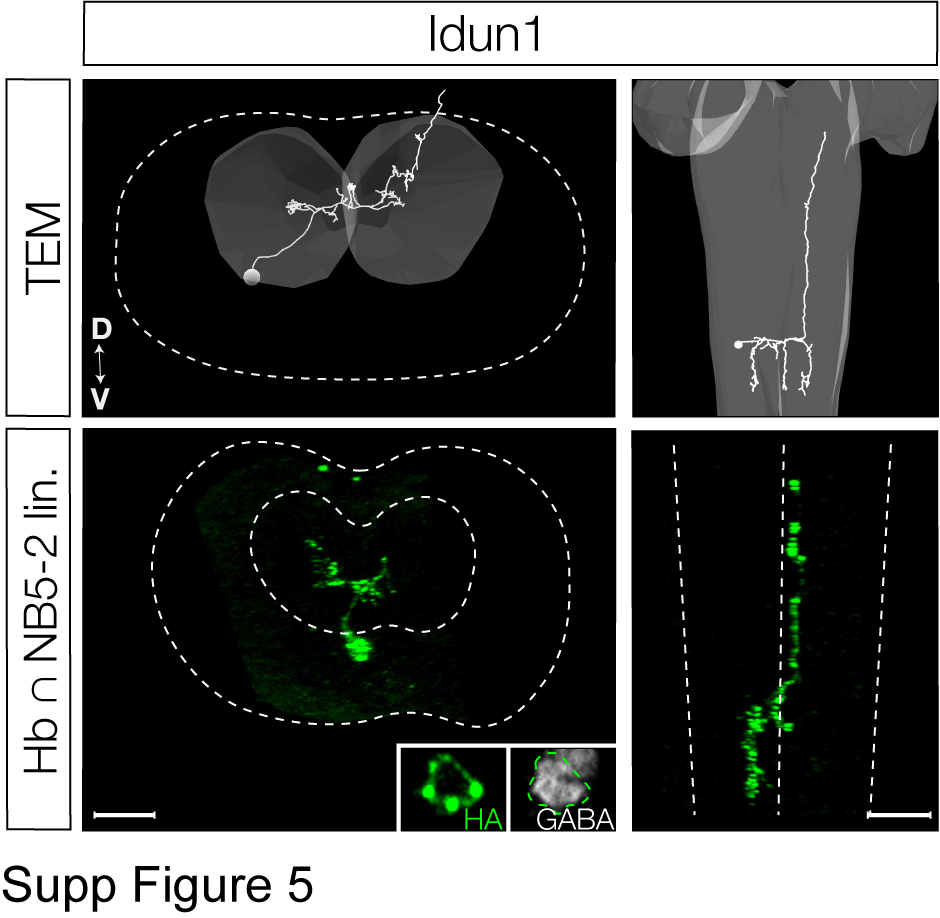

Supplement: S5 Fig — TEM reconstruction of Idun1 (upper panels) and single labeled Hb+ NB5-2 neuron genetically labeled with membrane-bound epitope tag, HA (green; bottom panels), with GABA expression (inset). Dorsal up, posterior view (left panels); Anterior up, ventral view (right panels). Scale bar: 10 µm. (TIF) [file pbio.3002881.s005.tif]

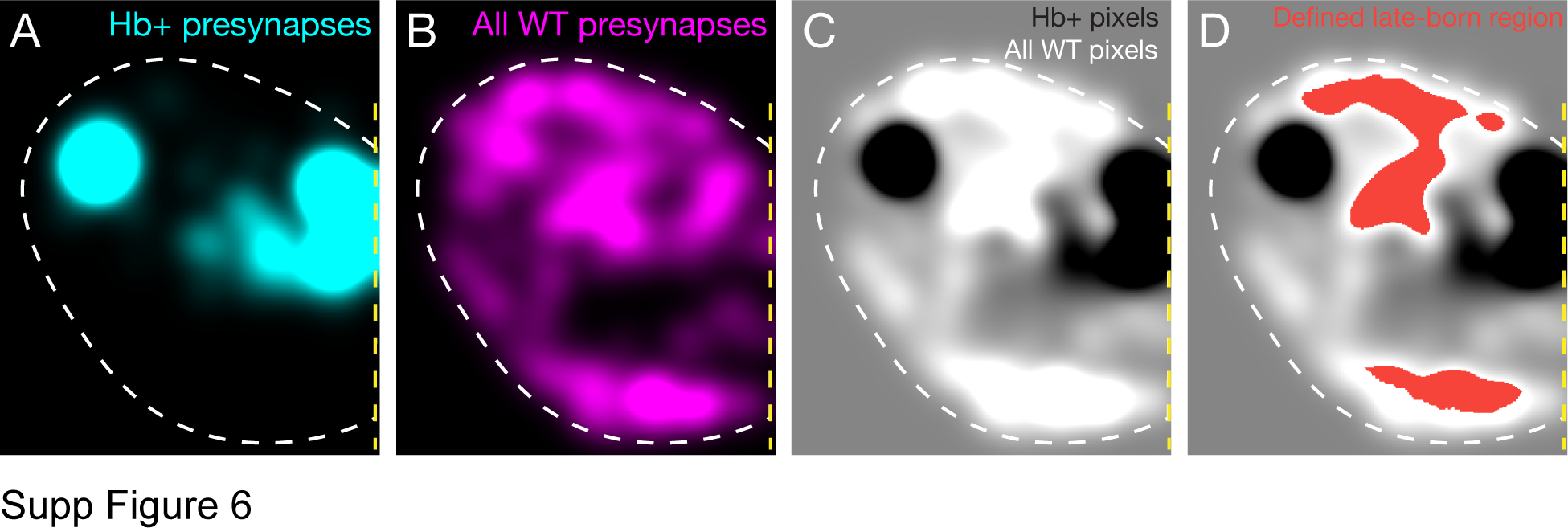

Supplement: S6 Fig — (A) Gaussian blur (1 μm) was applied to Hb+ NB5-2 presynapses (cyan; n = 17 hemisegments, 8 animals) after aligning to a template neuropil and normalizing intensity by a factor of 10/hemisegment. A single optical slice is shown. Dorsal up, posterior view. (B) Gaussian blur (1 μm) was applied to randomly selected WT NB5-2 presynapse images (magenta; n = 4 hemisegments, 2 animals) after aligning to a template neuropil and normalizing intensity by a factor of 1/hemisegment. Differing normalization factors between NB5-2 Hb+ and WT images were chosen to equalize intensities due to differences in presynapse number between NB5-2 Hb+ and WT images. (C) NB5-2 Hb+ regions (black) subtracted from WT NB5-2 presynapse regions (gray) to generate an image where pixel intensity corresponds to distance from late-born presynapses. (D) Pixels above a set threshold (red) define the late-born presynapse region. The pixel threshold was determined by setting the late-born template volume equal to the previously defined Hb+ presynapse volume. (TIF) [file pbio.3002881.s006.tif]

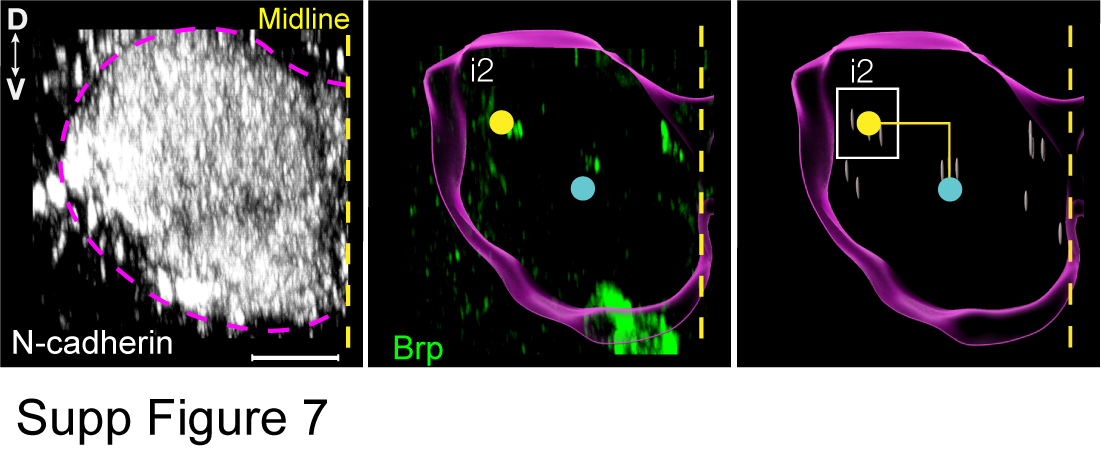

Supplement: S7 Fig — Neuropil labeled with N-cadherin (white) to define the neuropil border (magenta dotted line; left panel). The neuropil border (magenta) was defined using Imaris 10.0.1 surface tool to find the centroid (cyan dot) of a hemisegment and the center of Idun1–3 presynapse position labeled with Brp staining (green; middle panel). Shown is the Idun2 presynapse neuropil position (i2; yellow dot). The average i2 coordinate location was then found by measuring the dorsal-ventral and medial-lateral distance from the centroid (yellow solid line; right panel). The size of the presynapse volume (white box) was determined by the average distance from the centroid ± 2 standard deviations. Individual presynapses were quantified using the Imaris 10.0.1 spots tool (gray dots). Dorsal up, posterior view. Scale bar: 5 µm. (TIF) [file pbio.3002881.s007.tif]
