# Supplementary material for: The Hunchback transcription factor determines interneuron molecular identity, morphology, and presynapse targeting in the Drosophila NB5-2 lineage
Source: PLoS Biol. 2025 Mar 31;23(3):e3002881. doi: 10.1371/journal.pbio.3002881 (PMC12135938; doi:10.1371/journal.pbio.3002881)
Supplement: Attachment — Submitted filename: reviewer response.docx [file pbio.3002881.s018.docx]

Reviewers' comments

Rev. 1:
The manuscript by Pollington and Doe on the role of Hunchback in determining interneuron identity in the Drosophila MB5-2 lineage is truly extraordinarily impressive. They provide detailed and thorough evidence that support the idea that the cascade of temporal transcription factors, rather than birth timing or order, for example, is responsible for the molecular identity, morphology, and presynapse targeting of interneurons in this lineage. Previous work from the Doe group has made this point in a compelling way for certain Drosophila motoneurons, but interneurons have been far more challenging to study due to the number and diversity of these cells, and the lack of markers to identify them individually. The experiments here report a truly fabulous amount of work at a uniquely high level of resolution. It is a testament to the previous work of this group and a relatively small cohort of other labs that the interpretation reported here will probably not come as a huge
surprise in the field, but it is worth remembering that not that many years ago this whole notion would have been dismissed out of hand by many of those who were interested in the mechanisms underlying neuronal connectivity. That gives this work a great deal of significance in the larger context of developmental neuroscience.
Thank you for that accurate summary and positive comments.

I have few comments to make, and they are minor. I will confess that here and there one finds a couple of images where it is difficult for the non-expert to tell what distinguishes signal from nonspecific background in some cells of some micrographs, but I think this is quite unavoidable given the nature of the experiments. It does not cause me to question the quantification, or interpretations, of the authors. I also direct the authors to the paragraph of lines 398-405, where, unless I am mistaken, a sentence or two seem to be missing that would specify the phenotypic classes that are then discussed (though the point of the paragraph is clear and the caveats well-considered).
We thank you for bringing our attention to the signal to background noise of some images that may be difficult for non-experts to distinguish. Although we agree this was unavoidable for this study, we will keep this in mind for future studies.

We have also added a clarifying sentence in the paragraph of lines 420-424 defining each of the phenotypic classes.


Rev. 2:
Pollington and Doe address the role of temporal transcriptional changes in neural stem cells on the differentiation of their progeny. They focused on three major identity features: molecular identity, axon/dendrite morphology, and presynaptic targeting. In conclusion, their study clearly shows that one can transform late born neurons into having identities of early born neurons by forcing the expression of an early factor in aged neural stem cells.

Similar studies have been published by this group and others on different neuronal lineages in Drosophila embryos. This study is unique in terms of its completeness. The authors first introduce the NB5-2 lineage: identify temporal factors expressed in the neural stem cells, identify transcription factors expressed in the progeny, and visualize morphology and synaptic output sites at single neuron level. In addition to this, they performed the functional analysis and demonstrated that they can force late-born neurons to adapt early-born cell fate identity by simply misexpressing Hb in neural stem cells.

Thank you for the summary and commentary on the completeness of this study.

Although the study is documented well in this manuscript, minor revisions needed.

Figure 8 is not very convincing excluding the N2 region. The main reason is that the methodology is open for human errors at choosing the correct regions across different samples. Even within the same animal, left and right corresponding boxes don't match (e.g., N3 in Fig 8E). Depending on where you drove those boxes, you can get different results. Authors can keep the figure, but I suggest not making the conclusion for the N2/N3 regions.

Thanks for this comment, we agree that human bias and left/right asymmetry may result in excessive variability. To address this, we've made two changes. First, we use hemisegments as the unit of development, which is appropriate due to their developing independently. Second, and more importantly, in response to your comment we developed an Napari algorithm to register the Hb+ and Hb- presynaptic puncta (see methods). This code reduces human bias and standardizes presynapse analysis. This more rigorous approach confirms our previous approach, and we now report both methods in new Figure 8 and new Supplemental Figure 6.

Authors need to indicate how many cells in average they see for their driver at stage 14? Do the authors see all the 18 expected cells (maybe a few lower because of apoptotic progeny) in their NB5-2-GAL > GFP labeling? If not, do they think this might cause issues related to their conclusions about the composition and birth order and how can they address this?

We have quantified the number of cells at stage 14 and have added the data to Figure 2F and the corresponding text in lines 172-174.

Do we expect that the NB/GMC expression is inherited and maintained by both Notch OFF and OFF cell types? Is that the case in the previously established 6ages? Or is it possible both cells inherit the GMC marker, one cell later represses it as seen in the Eve+ RP2 and its Eve- sibling cell?

We do expect that the NB/GMC Hb expression is inherited and maintained by both NotchON/OFF cell types. We have quantified the number of Hey+ (Notch marker) NB5-2 neurons in stage 17 WT and Hb misexpressed embryos and found no significant difference (WT avg=10.2, Hb misexpression avg=10.25, n=16 hemisegments, 4 animals). The number of Hey+ neurons of each genotype represent approximately half the neurons in the lineage, consistent with the production of NotchON/OFF sibling pairs. In addition, previous work from our group has shown that NotchON neurons project to the dorsal neuropil domain and NotchOFF neurons project to the ventral domain. We find that Hb misexpression results in the production of both ventral (Idun1 and Idun3) and dorsal (Idun2) projecting late-born transformed neurons (see Figure 7) representing both NotchON/OFF neuron types.

Conclusion made in Fig3 can be drawn from Fig 2. Do authors consider merging them into one figure?

We feel that merging the figures would make a combined figure unwieldy and too complex. The data would not be any different either way, and currently the figures each have a corresponding Results section. However, we defer to the editor; if he feels that it is best to merge the two figures we will accept that choice.

Figure 4 clearly shows that ectopic Hb expression can repress later TTs. However, it is not clear whether they checked Kr, Pdm expression across all time points. I assume they did, and they found the last 6 division occurs from Hb only NBs till stage 14. Do authors think that Hb expression after the competence window (stage 12-14) is able to reprogram the NB to Hb window? Or NB5-2 has a different competence? It would be good if these issues are addressed.
We are sorry the figure was confusing, and we have altered it to be clearer. We did quantify the number of Kr+ and Pdm+ neurons (Supp Fig 2). In addition, we found an average of 3 Cas+ neurons in Hb misexpressed embryos suggesting that the Hb competence window for NB5-2 closes at approximately stage 13-14, during the WT Cas expression window.

Minor issues:
Figure 4 B WT Hb-Kr NB missing the Kr's blue color. It should be half/half but it is green only.

Thank you for your attention to detail! We have fixed the colors.

Figure 7: what stage embryos/ larvae used? If late-stage embryos used, this might explain the minor differences between light and EM morphology.

Thank you for providing a possible explanation. We used L1 (0-3hr ALH) larvae. TEM data was collected from a L1 (4-6hr ALH) larva; however, WT Idun1-3 neurons show strong ascending projections in 0-3hr ALH larvae (see Figure 6). We have added the larval age information to Figure 6B and Figure 7A and D legends for clarification.

Line 73-76: The author previously showed NB7-1 make a Nkx6+ Motor neuron in addition to U1-U5. It would be good to reiterate it here.

We agree, and have added VO as requested, lines 76-78.

Line 80-81: "early-born (late-born) muscle targets" implies muscles are born early but I am assuming author meant the neurons. Need to clarify.

Thank you for highlighting confusing wording. We have changed the wording for clarity, lines 82-83.

Materials: Dbx antibody source/dilution is missing.

Thanks! We have added Dbx antibody information to the “Immunostaining and imaging” section under Materials and Methods

Line 124-126: Cited references did not show that NB5-1 lineage does not have Hb, Kr, Pdm expression. Considering it delaminates at S5, it is not expected to express these factors but It would be nice if the authors provide evidence for this or change the language to indicate it is expected.

Thanks, we agree and now cite a paper (Cui and Doe, 1992) showing that NB5-1 delaminates as Cas+. It is highly unlikely to express earlier TTFs (Hb, Kr, Pdm) but this has never been determined.

Line 168-169: It would be good to show the second Cas window for the NB5-2 in figure 1 by extending the time scale to 16hr embryos. It would be also nice to prior publication(s) showing the second Cas window.

Interestingly, we find that NB5-2 does not have a second Castor window. We have adjusted Figure 2D to reflect one Cas window and quantified all NB5-2 Cas+ alone progeny at stage 14.

Line 251-254: The method section is missing the procedure to generate clones via hs-KD induction. When were the animals heat-shocked? during different stages of embryogenesis? Did they see over-represented clones of MN12/Idun 1 sibling cells assuming they hit the GMC?

We have added a section to the Materials and Methods explaining this approach. Given the continued expression of Hb in NB5-2 Hb+ progeny throughout embryogenesis, we heat-shocked embryos at stage 17 which avoided Hb+ GMCs. We observed a relatively even representation of all 5 Hb+ NB5-2 neurons.

Figure 2C-C''': Why don't we see more Cas negative dorsal cells? From the figure legend it is not clear if shown cells are part of a larger clone.

The image is a single focal plane through a 3D clone which does not encompass all the dorsal cells; we have added a low-mag inset showing more of the clone, including Cas- dorsal cells.

Is Fig 2E represent only the divisions until the end of stage 14?

Fig 2E represents NB5-2 divisions throughout embryogenesis. We have added the title “Entire NB5-2 lineage expression cascade” for clarity

Line 766: "NB5-2>Hb progeny Hb (top panels)" is not clear, is there typo here?
We meant to define the top row as NB5-2 expressing Hb and the bottom row showing expression of later TTFs in NB5-2 Hb misexpressed embryos. We have added text on line 876 to make the description of each row clearer.


Rev. 3: Tzumin Lee – note that this reviewer has signed his review
This solid study by the Doe lab is well done. It reports in great details the VNC 5-2 lineage that yields distinct interneurons per temporal transcription factors. It establishes another model lineage for in-depth mechanistic studies from neuronal temporal fates to neuronal differentiation and ultimately organism behavior. The genetics to target and label the VNC 5-2 lineage with single neuron resolution is elegant. The phenotypes of TF expressions and neuron anatomy in wild-type and perturbed conditions are clearly described with beautiful figures. Discussions are faithful and insightful. I support publication of the paper as it is.
Thank you for your kind comments and support for publication of this paper.


Rev. 4:
This manuscript presents a thorough analysis of the role that the temporal transcription factor Hb controls early fates in a lineage that had not been previously studied, and one that generates interneurons instead of motor neurons. For the most part, the genetic manipulations and data are unambiguous and in some cases elegantly designed. In particular, we appreciated the single neuron analysis and genetic labeling strategy used in Figures 6-8.

Thank you for your positive comments and appreciation for our single labeling approaches.

There are a few questions that should be resolved before publication:
There is ambiguity regarding the total number of Hb+ neurons. The text mentions 5 Hb+ neurons, whereas only 4 Hb+ neurons are visible in Figure 2A'.

Our Hb filtered genetics approach revealed there are 5 morphologically distinct Hb+ NB5-2 neurons. However, we do not see GFP expression driven by the NB5-2 Gal4 driver until embryonic stage 10, thus we believe that the first GMC division is not labeled with GFP in every embryo, which leads to an average quantification of 3-4 Hb+ NB5-2 neurons in stg 14 embryos. We have clarified this in the text on lines 166-167.

For Figures 1-4, please include the total number of replicates in each figure legend.

Thank you for pointing out this missing detail. We have added the number of replicates in the legends.

In Figure 5, the data suggest that the total number of neurons remains constant despite Hb overexpression in late-born neurons. To verify this observation, performing BrdU staining is recommended.

We have quantified the total number of NB5-2 progeny in WT and Hb misexpressed stage 17 embryos and did not find a significant difference in the number of neurons in the lineage (lines 236-237, see Fig 5X). While performing BrdU would strengthen this conclusion, we believe our quantifications are sufficient to show that Hb overexpression does not alter the number of neurons in the lineage.

Using neuronal settling position as a proxy for neuronal birth order may lead to misidentification of a larval interneuron. Calculating the relative distance of newly born neurons with respect to their neuroblast (NB) may provide more precise information on each neuron's birth order.

Thank you for suggesting an alternative approach. We use a combination of neuronal settling position and GFP labeling, driven by the NB5-2 Gal4, to approximate the birth order of NB5-2 neurons specifically. In this way, we are able to identify NB5-2 interneurons apart from other interneurons in the CNS.

The authors use the TEM dataset to locate ROIs for the presynapse analysis, but how confident can they be that there is a direct 1:1 correspondence between the TEM and light microscope images? Given that the neurites are changing length, could the ROIs be shifting, leading to errors in the presynapse analysis?

Good points! We do all analyses at the same larval stage, to avoid the inevitable changes over developmental time. You are correct in questioning whether there is a 1:1 relationship between TEM and LM analyses -- we agree and feel that it is unlikely. Importantly, the comparison is LM imaging of control and Hb OE larvae, which doesn't depend on a 1:1 relationship with LM and TEM data; in fact, the TEM data was only used to predict and confirm the position of Idun1-3 presynapses in the neuropil across animals, and not to compare the precise number of presynapses in the TEM volume compared to those in the LM volume.

Do the 5-2>Hb flies hatch? What happens to this lineage post-embryonically?

NB5>Hb flies do hatch and Figures 6-9 show morphological and pre-synapse targeting phenotypes in L1 (0-3hrs ALH) larvae. Many NB5-2>Hb larvae begin to die as they progress through larval life; however, we have observed morphological phenotypes in L3 larvae (72hr ALH). We did not look at lineage phenotypes in pupal or adult flies.
